# Adalimumab (tumor necrosis factor-blocker) reduces the expression of glial fibrillary acidic protein immunoreactivity increased by exogenous tumor necrosis factor alpha in an organotypic culture of porcine neuroretina

**Published:** 2013-04-17

**Authors:** I. Fernandez-Bueno, M.T. Garcia-Gutierrez, G.K. Srivastava, M.J. Gayoso, J.M. Gonzalo-Orden, J.C. Pastor

**Affiliations:** 1 Instituto Universitario de Oftalmobiología Aplicada (IOBA), University of Valladolid, Valladolid, Spain; 2 CIBER de Bioingenieria, Biomateriales y Nanomedicina (CIBER-BBN), Spain; 3 Regenerative Medicine and Cell Therapy Networking Center of “Castilla y León”, Spain.; 4 Departamento de Biología Celular, Histología y Farmacología; University of Valladolid, Valladolid, Spain; 5 Instituto de Biomedicina (IBIOMED), University of León, León, Spain

## Abstract

**Purpose:**

To determine if exogenous addition of tumor necrosis factor alpha (TNFα) exacerbates retinal reactive gliosis in an organotypic culture of porcine neuroretina and to evaluate if concomitant adalimumab, a TNF-blocker, diminishes it.

**Methods:**

Porcine retinal explants from 20 eyeballs were cultured. Cultures with 100 pg/ml TNFα, 10 µg/ml adalimumab, 100 pg/ml TNFα plus 10 µg/ml adalimumab, or controls without additives were maintained for 9 days. Freshly detached retinas were processed in parallel. TNFα levels in control culture supernatants were quantified with enzyme-linked immunosorbent assay. Cryostat sections were doubly immunostained for glial fibrillary acidic protein (GFAP), a marker for reactive gliosis, and cellular retinaldehyde-binding protein (CRALBP), a marker for Müller cells. Sections were also labeled with the isolectin IB_4_, a label for microglia/macrophages.

**Results:**

TNFα in control culture supernatants was detected only at day 1. Compared to the fresh neuroretinal samples, upregulation of GFAP and downregulation of CRALBP occurred during the 9 days of culture. Exogenous TNFα stimulated glial cells to upregulate GFAP and downregulate CRALBP immunoreactivity. TNFα-treated cultures also initiated the growth of gliotic membranes and underwent retinal disorganization. Adalimumab inhibited the spontaneous increases in GFAP and maintained CRALBP. In combination with TNFα, adalimumab reduced GFAP expression and conserved CRALBP, with only slight retinal disorganization. No appreciable changes in IB_4_ labeling were observed under the different culture conditions.

**Conclusions:**

In cultured porcine neuroretina, spontaneous reactive gliosis and retinal disorganization were exacerbated by exogenous TNFα. Adalimumab reduced spontaneous changes and those induced by TNFα. Therefore, inhibiting TNFα may represent a novel approach to controlling retinal fibrosis observed in some human diseases.

## Introduction

Proliferative vitreoretinopathy (PVR) is the main cause of failed rhegmatogenous retinal detachment (RD) surgery (approximately 5%–10% of cases) [1]. PVR is the result of an overstimulated wound healing process induced after a retinal break, and is characterized by marked fibrotic and inflammatory responses [2,3]. This process is likely initiated by a cascade of cytokines and growth factors produced by interactions between resident and non-resident retinal cells [3]; chief among them are glial cells and macrophages [3-5]. Glial cells, mainly Müller cells, strongly proliferate, form fibrocellular membranes, and induce intraretinal changes that characterize the most clinically severe forms of PVR [6-8]. Macrophages migrate into the retina after the breakdown of the blood–retinal barrier [9,10] and secrete several proinflammatory and proangiogenic cytokines, such as tumor necrosis factor alpha (TNFα). TNFα intraocular synthesis is increased in PVR [11-13], and TNFα binds to receptors on Müller cells and probably activates them [14,15]. Furthermore, microglia, the resident macrophages of the retina, become activated after retinal damage [16] and potentially release transiently high levels of TNFα [17]. TNFα also plays a significant role in various intraocular diseases such as uveitis, glaucoma, and retinal degenerations [18-20]. Therefore, regulating and suppressing TNFα using various biologic agents has recently emerged as a therapeutic strategy for several ocular inflammatory conditions [21-25].

Organotypic culture of the neural retina has been demonstrably useful for improving the knowledge of neurodegenerative disease pathophysiology. Several methods have been described for culturing retinal explants from different species. In the late 1980s, Caffe et al. [26] developed a method in which the neural retina is placed with the photoreceptor layer facing downward on rafts made of nitrocellulose filters and polyamide gauze grids. Since then, variations of this method have been used in several studies to evaluate the therapeutic effect and potential toxicity of substances [27-30]. Furthermore, retinal explant culture systems can mimic the functional dynamics of the organ beyond those of the dissociated cells [31], and many alterations observed during in vitro retina culturing [26,32-35] resemble some characteristics of experimental RD in vivo [36]. Thus, these similarities allow further research of pharmacological and bioengineering treatment modalities [37,38].

Interactions between glial cells and macrophages via TNFα could have a key role in the pathogenesis of PVR, and this cytokine could be a target for treating this disease. Adalimumab is a recombinant human monoclonal antibody specific for TNFα that forms stable bonds with this cytokine [24]. Adalimumab has been successfully used in treating systemic inflammation such as rheumatoid arthritis and Crohn’s disease [24], and ocular inflammation such as uveitis and Behcet’s disease [39,40]. Our group has experience in a model of organotypic culture of porcine neuroretina in which increased reactive gliosis modifications occur when retinas were cocultured with macrophages [35]. Thus, the purpose of this work was to determine if exogenous TNFα exacerbates retinal reactive gliosis modifications in an organotypic culture and to evaluate if concomitant adalimumab could diminish it. Experimental testing of new drugs in this field is necessary because previous medical approaches for treating PVR or inhibiting retinal reactive gliosis have already failed [2,8].

## Methods

### Tissue culture

Twenty fresh porcine eyes from animals aged 6–8 months old were obtained from the local slaughterhouse. Immediately after enucleation, the eyes were immersed in ice-cold transport medium composed of Dulbecco’s Modified Eagle Medium (DMEM) supplemented with a 1% antibiotic-antimycotic mixture containing penicillin, streptomycin, and amphotericin B (both Gibco, Paisley, UK) and transported on ice to the laboratory. Under aseptic conditions, each eyeball was immersed in 70% ethanol and then washed in clean DMEM. Neuroretinal explants were obtained as previously described by our group [35]. Briefly, the eyes were dissected to exclude the iris and the lens. The vitreous was then removed from the posterior eyecup with cotton swabs. The entire neuroretina was detached by paintbrushing and cutting the optic nerve. Finally, the neuroretina was unrolled in a Petri dish containing Neurobasal A medium (Gibco, Paisley, UK) supplemented with the 1% antibiotic-antimycotic mixture. The neuroretina was then cut into 5×5 mm explants in such a way as to avoid taking the most peripheral retina and visible blood vessels.

A total of 100 retinal pieces were obtained. Eighty were explanted on Transwell culture dishes (0.4 μm pore, 24 mm; Corning Inc., Corning, NY) with the photoreceptor layer facing the membrane. Explants were cultured in Neurobasal A medium supplemented with 2% B-27, 2% fetal porcine serum (both Gibco), 1% L-glutamine (Sigma-Aldrich, St. Louis, MO), and 1% antibiotic-antimycotic mixture. Explants were maintained at 37 °C in an atmosphere of 5% CO_2_ with 95% humidity. The culture medium level was maintained in contact with the support membrane beneath the explant and changed with freshly prepared warmed medium on the following day and then every second day. Explants were cultured in different experimental conditions described below, and harvested for analysis after 9 days. Twenty fresh neuroretinal samples were used as culture day 0 controls and processed in parallel.

### Experimental conditions

#### Control culture

To determine spontaneous retinal reactive gliosis modifications during culture, 20 neuroretinal explants were maintained in the culture medium described above.

#### Tumor necrosis factor alpha–treated culture

To determine if exogenous TNFα induced changes in retinal reactive gliosis, 20 neuroretinal explants were cultured with 100 pg/ml of porcine TNFα (*Escherichia coli* derived, R&D Systems, Minneapolis, MN) added to the medium at day 0. The cytokine concentration was based on previous studies in which the levels of TNFα produced by human monocytes were described [41,42] and in previous studies performed by our group (data not yet published).

#### Adalimumab-treated culture

To determine if adalimumab could block or diminish neuroretinal reactive gliosis modifications that occur spontaneously during culture, 20 neuroretinal explants were cultured with 10 µg/ml of adalimumab (Humira®, 40 mg/0.8 ml, Abbott Laboratories Ltd., Queenborough, UK) added to the culture medium at day 0. The adalimumab concentration was selected based on its efficacy in culture as described elsewhere [43,44].

#### Tumor necrosis factor alpha plus adalimumab-treated culture

To determine if adalimumab could block or diminish neuroretinal reactive gliosis modifications induced by exogenous TNFα, 20 neuroretinal explants were cultured with 100 pg/ml of TNFα plus 10 µg/ml of adalimumab added to the culture medium at day 0.

### Tumor necrosis factor alpha quantification

TNFα concentration was determined in the control culture supernatants collected at medium exchange, on days 1, 3, 5, 7, and 9 of culture (n=5 each). The concentration was measured with quantitative enzyme-linked immunosorbent assays specific for porcine TNFα (Quantikine; R&D Systems, Abingdon, UK). The mean minimum dose of porcine TNFα detected with this method was 3.7 pg/ml.

### Tissue processing

Samples were fixed in 4% paraformaldehyde (Panreac Química S.L.U., Barcelona, Spain) in phosphate buffer, pH 7.4, for 2 h and then subjected to sucrose cryoprotection [45]. On the following day, they were embedded in Tissue-Tek (O.C.T. Compound; Sakura Finetek Europe B.V., Alphen, the Netherlands). Sections (5 μm) were cut on a cryostat and mounted on glass slides (SuperFrost Plus; Menzel-Gläser, Braunschweig, Germany). Sections were doubly immunostained with primary antibodies against glial fibrillary acidic protein (GFAP, 1:500 rabbit polyclonal; DakoCytomation Inc., Glostrup, Denmark) as a reactive gliosis marker, and cellular retinaldehyde-binding protein (CRALBP, 137 1:1,000 mouse monoclonal [B2]; Abcam plc., Cambridge, UK) as a Müller cell functionality marker. Both antibodies were diluted in phosphate buffer containing 0.5% Triton X-100 (Sigma-Aldrich, St. Louis, MO), and incubated 30 min at room temperature for GFAP and overnight at 4°C for CRALBP. The next day, sections were washed in phosphate buffer. Thereafter, the corresponding species-specific secondary antibodies to immunoglobulin gamma conjugated to Alexa Fluor 488 (green) and/or 568 (red; both from Molecular Probes, Eugene, OR) were applied at a 1:200 dilution for 1 h. Some sections were incubated in 5% normal goat serum (Sigma Aldrich) in phosphate buffered saline (PBS; pH 7.4; Gibco), with 0.5% bovine serum albumin, 0.1% Triton X-100, and 0.1% sodium azide (Sigma-Aldrich), overnight at 4 °C. The following day, the isolectin IB_4_ (1:50 isolectin GS-IB4 from *Griffonia simplicifolia*, Alexa Fluor 488 conjugate; Molecular Probes) was added and incubated overnight at 4 °C. This lectin was used as a label for microglia/macrophages. Finally, cellular nuclei were stained with 10 μg/ml 4’,6-diamino-2-phenylindole dihydrochloride (Molecular Probes) for 8 min. Sections were washed in PBS (Gibco), mounted in Fluorescence Mounting Medium (DakoCytomation Inc., Carpinteria, CA) and coverslipped.

Fluorescence was detected with a Leica DM4000B light microscope equipped for epifluorescence (Leica Microsystems, Wetzlar, Germany), and images were obtained with a Leica DFC490. Comparative studies of immunoreactivity expression were performed on images acquired at the same levels of exposure, intensity, and gain. Brightness and contrast were finally adjusted using Adobe Photoshop 7.0 (Adobe Systems, San Jose, CA). Primary antibodies used in this work were used in previous studies and are well characterized by us and other authors regarding specific cell-type immunostaining. Furthermore, control slides in which primary antibodies were omitted were processed in parallel, with no immunoreactivity found in any case.

## Results

### Fresh neuroretinal samples

**Figure 1:**
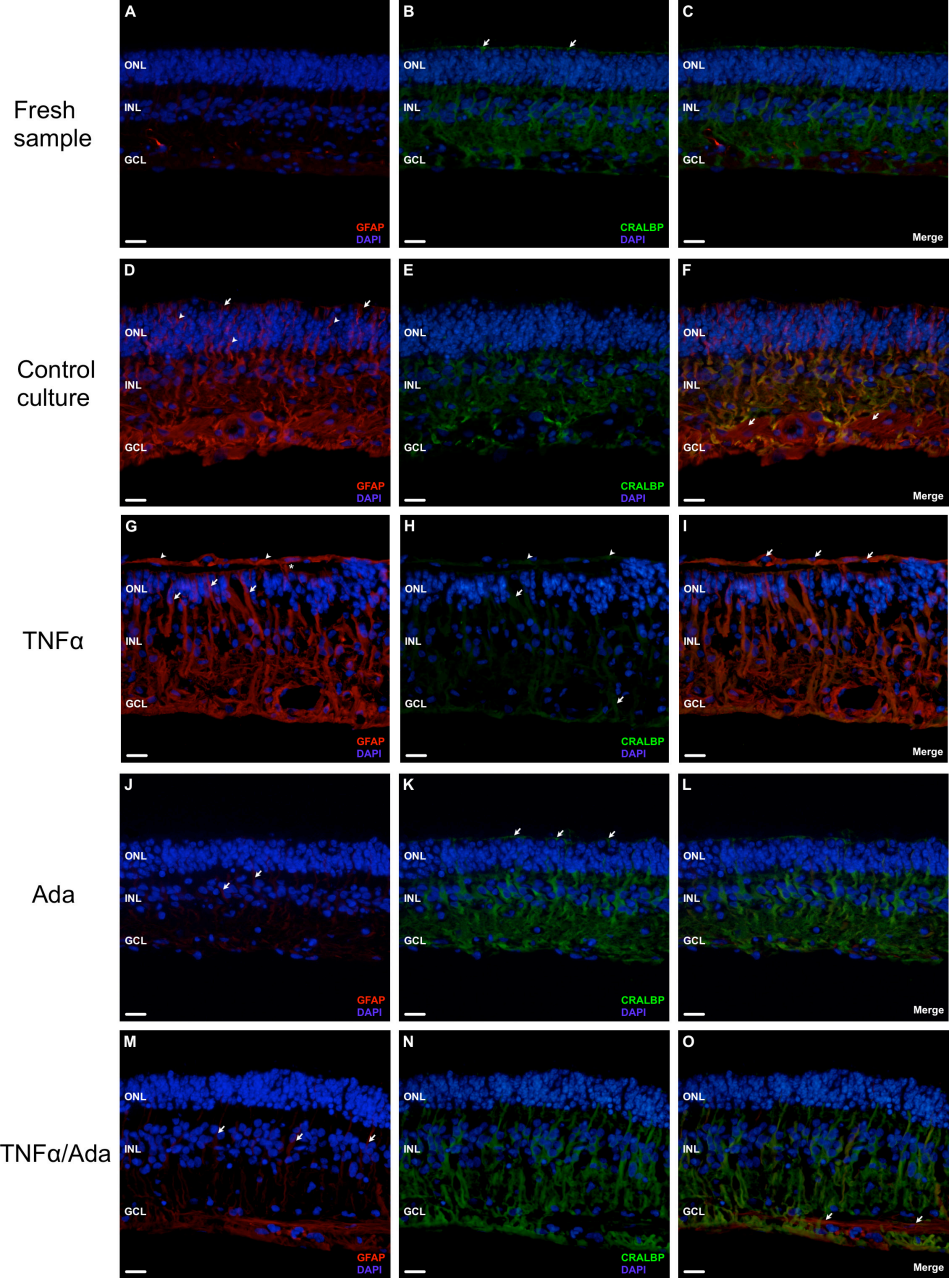
Retinal distribution of glial fibrillary acidic protein (GFAP, red; **A**, **D**, **G**, **J**, **M**) and cellular retinaldehyde-binding protein (CRALBP, green; **B**, **E**, **H**, **K**, **N**), and corresponding merged compositions (Merge; **C**, **F**, **I**, **L**, **O**), in fresh neuroretinal samples (**A**–**C**) and experimental 9 day culture conditions (**D**–**O**). Ada: adalimumab treated culture; DAPI: 4’,6-diamino-2-phenylindole dihydrochloride staining (blue); INL: inner nuclear layer; GCL: ganglion cell layer; ONL: outer nuclear layer; TNFα: tumor necrosis factor alpha treated culture; TNFα/Ada: tumor necrosis factor alpha plus adalimumab treated culture. 4’,6-diamino-2-phenylindole dihydrochloride (DAPI) staining (blue) was present in the nuclei of the ganglion cell layer (GCL), the inner nuclear layer (INL), and the outer nuclear layer (ONL). In fresh specimens, glial fibrillary acidic protein (GFAP) expression was present in glial cells in the inner retinal layers (**A**). Cellular retinaldehyde-binding protein (CRALBP) was localized to the cytoplasm and extensions of the Müller cells (**B**), clearly visible along the outer limiting membrane (OLM; **B**, arrows). Retinal structure and cellular organization were adequately preserved before culturing. In the control cultures (**D–F**), GFAP expression increased (**D**) in the Müller cells and astrocytes (**F**, arrows). Müller cell branches at the ONL (**D**, arrowheads) and at the OLM (**D**, arrows) expressed GFAP; whereas CRALBP was reduced (**E**). Retinal tissue started to lose its characteristic organization. In cultures with tumor necrosis factor alpha (TNFα; **G–I**), GFAP was markedly upregulated (**G**). It appeared in Müller cell processes at the ONL (**G**, arrows) and crossing the OLM (**G**, asterisk). CRALBP was scarcely present in some Müller cells (**H**, arrows). Retinal cell nuclei were reduced and randomly distributed. Glial branches formed a layered structure, positive to anti-GFAP and anti-CRALBP markers (**G** and **H**, arrowheads). Cellular nuclei appeared along these membranes (**I**, arrows). Adalimumab (Ada) treatment (**J–L**) caused a reduction in GFAP expression (**J**) compared to control cultures (**D**). A few GFAP spots appeared in some Müller cells at the INL (**J**, arrows). CRALBP labeling appeared throughout the entire Müller cells to the OLM (**K**, arrows). Retinal structure and nuclei organization were preserved. TNFα cultures simultaneously treated with adalimumab (TNFα/Ada; **M–O**), showed GFAP upregulation in astrocytes (**O**, arrows). Whereas GFAP spots appeared in the cytoplasm of Müller cells into the INL (**M** arrows), CRALBP was present mainly at the inner layers (**N**). Scarce retinal disorganization was apparent. Scale bars: 20 µm.

**Figure 2:**
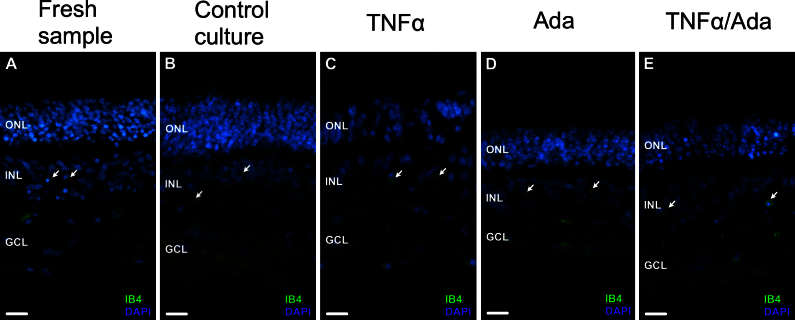
Retinal cells labeled with the lectin IB_4_ from Griffonia simplicifolia (IB_4_, green) in fresh neuroretinal samples (**A**) and experimental 9-day cultures (**B**–E). Ada: adalimumab treated culture; DAPI: 4’,6-diamino-2-phenylindole dihydrochloride staining (blue); INL: inner nuclear layer; GCL: ganglion cell layer; ONL: outer nuclear layer; TNFα: tumor necrosis factor alpha treated culture; TNFα/Ada: tumor necrosis factor alpha plus adalimumab treated culture. 4’,6-diamino-2-phenylindole dihydrochloride (DAPI) staining (blue) was present in the nuclei of the ganglion cell layer (GCL), the inner nuclear layer (INL), and the outer nuclear layer (ONL). IB_4_-labeled cells were present between the INL and the GCL and extended into the INL in the fresh samples and the 9-day culture experiments (**A–E**, arrows). Scale bars: 20 µm.

In freshly prepared specimens (0 day control; Figure 1A–C), GFAP immunoreactivity was localized to glial cells, astrocytes and Müller cells as confirmed with evaluation of cellular morphology. GFAP expression was limited to the innermost layers of the neuroretinal tissue (Figure 1A). CRALBP was present in the cell bodies and extensions of the Müller cells throughout the entire retina (Figure 1B). CRALBP was also evident along the outer limiting membrane (OLM; Figure 1B, arrows). 4’,6-diamino-2-phenylindole dihydrochloride staining of the nuclei allowed assessment of the ganglion cell layer (GCL), inner nuclear layer (INL), and outer nuclear layer (ONL) organization. IB_4_-labeled cells, typical of microglia and macrophages, were present in the retinal inner layers to the INL (Figure 2A, arrows). The complex retinal architecture was well preserved after mechanical detachment for explants preparation.

### Control culture

At 9 days of culture (Figure 1D–F), GFAP and CRALBP immunoreactivity was modified compared to the fresh samples (Figure 1A–C). GFAP was clearly upregulated (Figure 1D). In the inner layers of the retina, expression of this protein was mainly localized to astrocytes, as confirmed by cellular morphology and the absence of CRALBP labeling (Figure 1F, arrows). In the Müller cells, GFAP immunoreactivity extended from the end-feet through the cell body, into the outer retinal layers and the OLM (Figure 1D, arrows), whereas CRALBP labeling was reduced. CRALBP remained in the inner retinal layers, and it was not detected in the outer layers (Figure 1E). Furthermore, retinal tissue showed some disorganization, and the retinal cells were less densely packed at the ONL and the INL. Müller cell branches located between photoreceptor cell bodies expressed GFAP (Figure 1D, arrowheads). IB_4_-immunoreactive cells were apparent between the INL and the GCL and extended into the INL (Figure 2B, arrows).

The supernatants of cultured neuroretinas contained low, but detectable, levels of TNFα at day 1 (Table 1). After 3 days in culture and at all later stages, the concentration was below the detectable level.

**Table 1:** Porcine tumor necrosis factor alpha (TNFα) levels in culture supernatants from control organotypic neuroretina cultures as determined by enzyme-linked immunosorbent assay.

**Days of culture**	**TNFα % detected**	**TNFα (pg/ml)** **(mean ± SD)**
1	100%	4.2±0.4
3	0%	<3.7
5	0%	<3.7
7	0%	<3.7
9	0%	<3.7

TNFα mean minimun detectable dose is 3.7 pg/ml. SD: standard deviation.

### Effect of exogenous tumor necrosis factor alpha on cultured neuroretinas

After 9 days of culture, the retinal explants exposed to exogenous TNFα (Figure 1G–I) showed marked upregulation of GFAP with the cell bodies and processes of the Müller cells (Figure 1G). CRALBP expression was nearly absent, detectable only in some Müller cell bodies (Figure 1H, arrows). There were fewer retinal cell nuclei, and they were randomly distributed and surrounded by numerous GFAP-rich Müller cell processes. An increased number of these branches were located in the ONL (Figure 1G, arrows), disrupting photoreceptor organization, and some crossed the OLM (Figure 1G, asterisk). These processes extended outside the retinal tissue and formed a layered structure that stained with anti-GFAP and -CRALBP markers (Figure 1G, H, arrowheads). Furthermore, some cellular nuclei were distributed along these membranes (Figure 1I, arrows). Cells labeled with the lectin IB_4_ were present in the inner retinal layers to the INL (Figure 2C, arrows).

### Effect of adalimumab on cultured neuroretinas

In cultures treated with adalimumab (Figure 1J–L), glial cell expression of GFAP (Figure 1J) was comparable to the fresh samples (Figure 1A) and notably reduced compared with the control group (Figure 1D). Nevertheless, minor GFAP spots appeared in some Müller cell bodies at the INL (Figure 1J, arrows). CRALBP labeling remained throughout the entire cytoplasm of the Müller cells, extending to the OLM (Figure 1K, arrows). IB_4_-immunoreactive cells were detectable in the GCL and the INL (Figure 2D, arrows). The retinal structure and organization of the nuclei were well preserved after 9 days of culture.

### Effect of tumor necrosis factor alpha plus adalimumab on cultured neuroretinas

In the neuroretina explants exposed to TNFα and simultaneously treated with adalimumab (Figure 1M–O), GFAP immunoreactivity was increased in astrocytes, identified by morphology and the absence of CRALBP expression (Figure 1O, arrows). Müller cells showed spots of anti-GFAP marker within the cell bodies that extended into the INL (Figure 1M, arrows). CRALBP expression was absent at the outermost retinal layers but still present at the inner ones (Figure 1N). IB_4_-labeled cells were present between the INL and the GCL and extended into the INL (Figure 2E, arrows). There was some retinal disorganization, and the cellular nuclei were slightly less densely packed at the INL.

## Discussion

PVR is an anomalous scarring process related to ocular inflammation that occurs after RD [2,6]. Müller cells and macrophages seem to play an important role in its pathogenesis [6,46,47]. Cytokines potentially secreted by macrophages are implicated in PVR development, and TNFα, a proinflammatory cytokine, is considered a major effector [11-13,48]. In fact, our group previously reported increased reactive gliosis modifications in retinas cocultured with macrophages [35]. However, in cocultures of porcine retina with macrophages that did not produce significant TNFα levels, there were no appreciable retinal gliotic changes [49]. Therefore, TNFα could act as a signaling molecule, initiating the reactive response of the glial cells. For this reason, inhibition of TNFα may be a new therapeutic strategy in retinal fibrosis prophylaxis.

Organotypic culture of the neural retina is an adequate tool for reproducing some of the cellular dynamics after RD [26,32-34]. There are some obvious limitations of this culture system such as the axotomy of ganglion cells, the absence of retinal and choroidal blood supply, and the absence of the retinal pigment epithelium. Nevertheless, the morphology and functionality of the organ are temporarily retained, and experimental conditions are under control [31]. Therefore, we consider neuroretinal organotypic cultures a good model to further develop our understanding of the roles played by different retinal cells and cell signaling in the development of retinal degeneration.

In the current study, the characterization of fresh neuroretinal explants and retinal modifications during culture were consistent with previous studies [50,51] and in vivo models of RD [36,51]. Furthermore, spontaneous and transient production of TNFα by retinal cells detected in these experiments were also reported in organotypic cultures of the rat retina [17] and attributed to secretion by microglia. Retinal modifications observed after TNFα addition resulted in greater hypertrophy of glial cells and a higher level of retinal disorganization. Furthermore, the processes of Müller cells crossed the OLM and formed gliotic membranes in the subretinal space. Similar observations also occur in PVR [6,46] and have been described by our group when neuroretinas were cultured with macrophages [35]. Therefore, the secretion of TNFα by non-resident macrophages may be an integral part of the glial cell response in reactive gliosis and subretinal membrane formation. Thus, TNFα could be an important therapeutic target for PVR that has not yet been adequately explored.

The adalimumab concentration used in these experiments (10 µg/ml) was lower than the doses reported toxic after intravitreal injection in rabbits (5 mg/ml) [52,53], and were higher than the effective doses necessary to neutralize 90% of TNFα [54]. In adalimumab-treated cultures, hypertrophy of glial cells was not evident, and retinal organization and Müller cell functionality related to CRALBP expression were preserved. This finding, which involves the inhibition of TNFα spontaneously produced by retinal cells, emphasizes the important molecular role of this cytokine in the development of retinal reactive gliosis. Cultures treated with TNFα and adalimumab showed only the initial steps of glial cell modifications and slight retinal disorganization. Therefore, adalimumab considerably diminished TNFα-induced reactive gliosis and retinal degeneration during culture.

Microglia activation with subsequent cellular migration to the photoreceptor layer occurs after retinal damage [16]. In the present study, IB_4_-labeled cells, presumably microglia, showed a similar distribution through the retinal tissue in the fresh samples and under different culture experiments. In rat retinal cultures, activated microglia modifications were no longer observed after 7 days [17], which is in concordance with our findings at 9 days of porcine retina culture. Evaluation of retinal microglia dynamics was not the main purpose of this study, and further research will be necessary to discern the contribution to retinal gliosis of these cells and the cytokines released by the cells.

Numerous drugs have been tested to inhibit cell proliferation, membrane formation, and further contraction in animal models and cell cultures [8]. However, many have potentially severe side effects, and only a few have been used in clinical trials [2]. Recent promising studies have described a reduction in glial cell growth by an inhibitor of the protein kinase B/mammalian target of rapamycin pathway [55] or an inhibitor of the Rho-associated protein kinase pathway [30]. Nevertheless, the precise mechanism of action of both substances is unknown, and they have not been approved by the U.S. Food and Drug Administration.

In summary, our current data showed that adding exogenous TNFα to porcine neuroretina cultures upregulates GFAP expression with downregulation of CRALBP. Adding exogenous TNFα also induced the disorganization of retinal structure and formation of gliotic membranes. These changes are typical of retinal reactive gliosis processes [5]. Adalimumab diminished TNFα-induced modifications and contributed to preserving retinal organization. Thus, the data presented here suggest that adalimumab is an effective agent for decreasing glial cell modifications and retinal degeneration induced by TNFα, and therefore may represent a novel way to control retinal gliosis. Even though further studies are necessary, this represents an important step toward the potential clinical application of this TNF-blocker in retinal degeneration diseases.
